# Size of Left Cardiac Chambers Correlates with Cerebral Microembolic Load in Open Heart Operations

**DOI:** 10.4061/2010/143679

**Published:** 2010-06-13

**Authors:** Elena Z. Golukhova, Anna G. Polunina, Svetlana V. Zhuravleva, Natalia P. Lefterova, Alexey V. Begachev

**Affiliations:** ^1^A. N. Bakulev Scientific Center of Cardiovascular Surgery, Russian Academy of Medical Sciences, Moscow 119571, Russia; ^2^Anaesthesiology and Intensive Care Department, Medical Center of the State Bank of Russia, Moscow 117593, Russia

## Abstract

*Background*. Microemboli are a widely recognized etiological factor of cerebral complications in cardiac surgery patients. The present study was aimed to determine if size of left cardiac chambers relates to cerebral microembolic load in open heart operations. *Methods*. Thirty patients participated in the study. Echocardiography was performed in 2-3 days before surgery. A transcranial Doppler system was used for registering intraoperative microemboli. *Results*. Preoperative left atrium and left ventricular end-systolic and end-diastolic sizes significantly correlated with intraoperative microembolic load (*r*s = 0.48, 0.57 and 0.53, *P*s < .01, resp.). The associations between left ventricular diameters and number of cerebral microemboli remained significant when cardiopulmonary bypass time was included as a covariate into the analysis. *Conclusions*. The present results demonstrate that increased size of left heart chambers is an influential risk factor for elevated cerebral microembolic load during open heart operations. Mini-invasive surgery and carbon dioxide insufflation into wound cavity may be considered as neuroprotective approaches in patients with high risk of cerebral microembolism.

## 1. Introduction

Microemboli are a widely recognized etiological factor of cerebral complications in cardiac surgery patients. Our group found significant associations between microembolic load and deliria and cognitive dysfunction after on-pump operations [1, 2]. Microemboli are observed during on-pump operations in nearly all patients [1, 3]. However, the number of microemboli considerably varies in different cases being the largest in open heart operations. In the study of Abu-Omar et al. [4], open heart procedures were associated with a 22-fold increase in microemboli in comparison to off-pump coronary patients. Prevalence of postoperative delirium and cognitive dysfunction was reported to be considerably higher after valve operations in comparison with coronary surgery as well [1, 5]. 

Progress in on-pump technologies resulted in reduced cerebral microembolic load and, therefore, neurological complications during contemporary cardiac operations. Membrane oxygenators instead of bubble ones and arterial line filters are used at present in the majority of cardiac surgery centers. Off-pump surgery, epiaortic scanning, minimized aortic manipulations, and perfusionist interventions were introduced as emboli lowing approaches. Hence, correct knowledge about potential sources of microemboli increases quality of surgery and, therefore, improves outcomes. However, it is still unclear why the variability of cerebral microembolic load is so extensive in patients, which are operated on by the same surgical team using the same cardiopulmonary bypass circuit and surgical technologies. The large variability of intraoperative microembolic load in spite of the similar surgical methodology implicates unknown patient characteristics as possible contributing factors to the quantity of intraoperative microembolism.

The majority of microemboli occurring during cardiac surgery are air bubbles [4]. Air enters the circulation through open cardiac chambers, at initiation of cardiopulmonary bypass (CPB), through the bypass circuit after blood sampling and injection of drugs [6, 7], through veins in the operative field, and injections into central venous lines. Venous microemboli enter the systemic circulation by paradoxic embolism through a patent foramen ovale (present in up to 20%–35% of healthy population) or after transpulmonary passage [8]. 

Air tends to accumulate in the highest parts of the heart including left ventricular apex, left atrial appendix, and the upper wall of the left atrium [9]. Air bubbles are mobilized into the arterial bed not only during and after the heart resumes function and CPB is stopped. It was shown that such surgical manipulations as lifting the heart to visualize posterior anastomoses in on-pump and even off-pump coronary operations are accompanied by increased microembolic load to the cerebral circulation [3, 10]. It is logical to hypothesize that enlarged cardiac chambers would accumulate larger volumes of air which would reach systemic circulation during surgical manipulations and restoration of heart beating. The present study was aimed to determine if size of left cardiac chambers relates to cerebral microembolic load in open heart operations.

## 2. Materials and Methods

### 2.1. Patients

The present study protocols were reviewed and approved by the academic council of Bakulev's cardiovascular surgery center. The study design was explained to patients, and each patient gave an informed consent to participate. Inclusion criteria were, age 16–69 years old, preoperative cardiac ejection fraction >40%; and availability of middle cerebral arteries (MCAs) to be insonated through the transtemporal windows. Exclusion criteria were: emergency and reoperative surgical procedures, previous cerebrovascular accident, and serious concomitant non-cardiac disease. A total of 30 patients were included into the study. The patients underwent operations on aortic and/or mitral valve, with or without concurrent CABG. The preoperative and intraoperative characteristics of patients are presented in Table 1.

### 2.2. Anesthesia, CPB, and Surgical Management

The protocols of anesthesia and surgical techniques were standardized. Diazepam and morphine served as premedication. Anesthesia was induced and maintained with propofol, fentanyl, and pancuronium. The perfusion apparatus consisted of the Stökert S3 roller pump (Germany), DIDECO-703 membrane oxygenator (Italy), and a 40 *μ*m arterial filter. Nonpulsatile pump flow rates were maintained between 2.4 and 2.6 L ∗ min^−1^  ∗ m^−2^, and mean perfusion pressure at 60 mm Hg. The operations were accomplished during moderate hypothermia (28°C). All patients underwent median sternotomy, the aorta was cross-clamped, and the heart was arrested with anterograde cold pharmacological cardioplegia by solution of custodioli. Topical ice saline was used as an adjuvant to myocardial protection.

### 2.3. Echocardiography

Echocardiography was performed with a Hewlett Packard Sonos 5500 ultrasound system with a 4 MHz transducer. Each patient underwent echocardiographic assessment in 2-3 days before surgery. Subjects were studied with M-mode and two-dimensional echocardiography. Left atrium diameter was measured in the parasternal long axis view from trailing edge of the posterior aortic root—anterior left atrium complex to the posterior left atrium wall. Left ventricular end diastolic and systolic dimensions were determined from standard M mode measurements. Absolute values of cardiac dimensions were not normalized.

### 2.4. Intraoperative Transcranial Doppler Monitoring

A 2-MHz Transcranial Doppler (TCD) system (ANGIODIN, BIOSS, Russia) was used for continuous bilateral monitoring of middle cerebral artery blood flow (depth, 45 to 60 mm) during the operation. The probes were fixed transtemporally by a head brace. Registration of microembolic signals was conducted from the initiation to complete discontinuation of CPB. MEs were defined as transient, short-duration (<300 ms), high-amplitude signals with intensity of >5 dB higher than background noise and which were accompanied by a characteristic high-frequency “click”. Settings for ME detection were highpass filter—120 Hz, lowpass filter—1100 Hz, dynamic range—96 dB, temporal overlap—60%, and sample volume—10 mm. The stored raw data were revised rigorously after the surgery to verify the number of intraoperative ME. The summarized value of microembolic load was included into the analysis.

### 2.5. Statistical Analysis

Statistical analysis was performed using SPSS software for windows (SPSS 13.0, Chicago, IL, USA). The data were tested for normality with the Kolmogorov-Smirnov test and found to be normally distributed. Correlation analyses were conducted using bivariate and partial Pearson's tests. The correlations were considered significant at a probability level of *P *<  .05. The linear regression analyses were performed in order to evaluate the combined effects of several variables of interest on the variance of microembolic load. Predictor variables were entered into a simultaneous regression model.

## 3. Results

**Figure 1:**
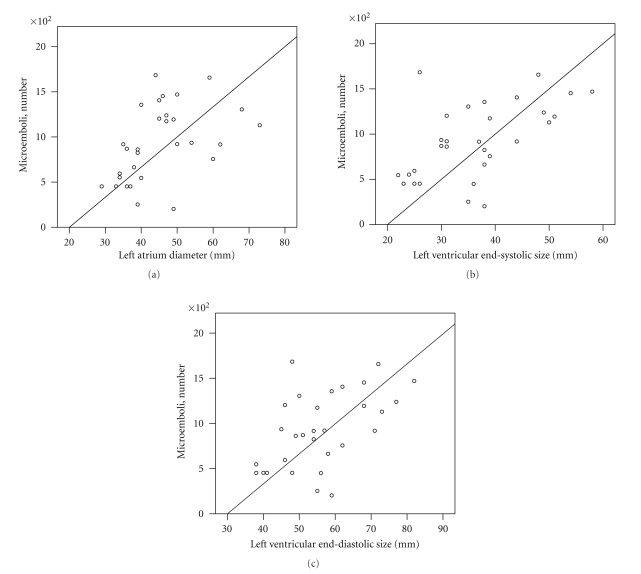
Left cardiac chambers size and microembolic load in open heart operations (*r*s = 0.48 −0.57, *P*s < 0.01). (a) Left atrium diameter; (b) left ventricular end-systolic diameter; (c) left ventricular end-diastolic diameter.

Preoperative left atrium diameter and left ventricular end-systolic and end-diastolic sizes significantly correlated with intraoperative microembolic load in open heart operations (*r*s = 0.48, 0.57 and 0.53, *p*s < .01, resp.) (Figure 1). As CPB time was significantly associated with both cardiac chambers size and number of microemboli, the former variable was included into the partial correlation tests as a covariate. The correlations between left ventricular end-systolic and end-diastolic sizes and microembolic load remained significant at partial correlation tests (*r*s = 0.42 and 0.43, *p*s < .05), whereas correlation between left atrium diameter and microemboli did not reach significance (*r* = 0.32, *P* =  .08). When any of left ventricular sizes was included into the regression model along with CPB time, both variables explained 42% of variance of cerebral microembolic load during open heart operations.

## 4. Discussion

The present results demonstrate that increased size of left heart chambers is an influential preoperative risk factor for elevated cerebral microembolic load during open heart operations. Although patients with enlarged hearts underwent longer CPB, the effects of cardiac size on cerebral microemboli were independent from CPB time. It is important to note that we studied the total number of intraoperative microemboli which entered the cerebral circulation during the whole CPB period. Nevertheless, cardiac size explained the considerable proportion of variance of intraoperative microembolic load in our patient cohort. Our findings implicate discussion of two important aspects: if gaseous microembolism is neurologically significant and contemporary approaches to prevention of air microembolism in open heart surgery.

### 4.1. Gaseous Microembolism and Neurological Injury

We used Transcranial Doppler system for registration of cerebral microemboli. The sensitivity of Doppler technology to gaseous microbubbles is much higher in comparison with particulate emboli [11]; therefore, the majority of microemboli registered at middle cerebral arteries in our patient cohort were air bubbles. Although many of cardiac surgery professionals consider gaseous embolism as a less important factor in comparison with particulate emboli, experimental and clinical data do not confirm this opinion. In the study of Martens and colleagues [12] bolus intracarotid injection of either air or CO_2_ induced multiple ischemic lesions in both homolateral and heterolateral brain hemispheres in experimental animals. Air bubbles occluded vessels and induced multiple small nonperfused areas in porcine retina in the study of Herren and colleagues [13].

In our relatively young patient cohort we found linear associations between intracerebral microembolic load to left hemisphere and postoperative verbal memory decline, and between microemboli to right hemisphere and nonverbal memory decline [1]. In the same patient population, the threshold association between microembolic load and postoperative delirium was determined; that is, patients with delirium were characterized by more than 900 microembolic signals at both MCAs during on-pump period [2]. It is interesting that Rudolph and colleagues [14] did not find significant association between cerebral microembolic load and delirium after coronary artery bypass graft surgery. This discrepancy between results of two studies may be explained by different approaches to diagnosis of delirium with more strict criteria in our study and more inclusive diagnostic criteria in the study of Rudolph et al. [14]. Older age and high prevalence of cerebral atherosclerosis were other possible contributing factors to the development of delirium in patients after coronary surgery in the study of Rudolph et al. [14]. The mean number of microemboli was about 300 in the latter study; nevertheless, 48% of patients demonstrated acute encephalopathy after surgery. It may be suggested that even relatively small number of cerebral microemboli is damaging in old atherosclerotic patients. 

Overall, the cited experimental and clinical studies evidence that intraoperative gaseous embolism is an important neuropathogenic factor. Although air microembolism is a routine event during cardiac and noncardiac operations [15], it should be prevented as thoroughly as possible, because the total number of microemboli is a linear correlate of brain damage [1, 16].

### 4.2. Prevention of Air Embolism in Open Heart Surgery

Intraoperative cerebral microembolism occurs not only during on-pump procedures; however, any surgical manipulation and even peripheral vein puncture induce air entrapment into systemic and cerebral circulation [15]. Therefore, mini-invasive surgical technologies with avoidance of opening heart chambers and cardiopulmonary bypass and minimized surgical manipulations are the important perspective of air embolism decrease in cardiac surgery. 

Deairing technologies are actively investigated at present as well [9, 17, 18]. Carbon dioxide insufflation into wound cavity appears to be neuroprotective. Martens and colleagues [18] demonstrated better neurological outcomes in CO_2_ insufflation group in comparison with control patients. The same research group found significantly more pronounced ischemic brain damage in pigs after intracarotid air injection in comparison with animals after intracarotid carbon dioxid injection [12].

Cardiopulmonary bypass with a prime of perfluorocarbon emulsions was shown to reduce air embolism in experimental studies [13, 19]. However, de Lange and colleagues [20] found excessive release of cytokines and higher mortality in animals primed with perfluorocarbon emulsions. 

In conclusion, patients with enlarged left cardiac chambers are at high risk of cerebral air embolism during open heart surgery. Prevention of air embolism is of importance as intraoperative microembolic load linearly correlates with brain damage. Mini-invasive surgery and carbon dioxide insufflation into wound cavity may be introduced as neuroprotective technologies in cardiac operations. Other embolism lowing methods need further research.

## Figures and Tables

**Table 1:** Patient characteristics.

Number of patients	30
Male sex (*n*/%)	15/50
Age (years)	44.3 ± 14.0
Height, cm	170.8 ± 8.3
Body weight, kg	81.4 ± 11.6
Body surface area, m^2^	1.82 ± 0.19
History of hypertension(*n*/%)	18/60
History of diabetes (*n*/%)	1/10
Previous myocardial infarction (*n*/%)	3/10
History of atrium fibrillation (*n*/%)	15/50
NYHA Class III and IV (*n*/%)	12/40
Left atrium diameter, mm	44.9 ± 10.7
Left ventricular end-systolic diameter, mm	36.2 ± 9.8
Left ventricular end-diastolic diameter, mm	55.5 ± 11.9
Mitral valve surgery (*n*/%)	17/57
Aortic valve surgery (*n*/%)	9/30
Aortic and mitral valve surgery (*n*/%)	4/13
CABG surgery (*n*/%)	4/13
Coronary artery bypass grafts (number)	3.1 ± 0.5
Cardio-pulmonary bypass time (min)	127.5 ± 59.2
Aortic cross-clamp time (min)	74.6 ± 39.2
Microembolic load (number)	914.6 ± 423.1

Data given as means ± SD, or as number of patients/% of patients. CABG*: *coronary artery bypass grafting; NYHA*: *New York Heart Association.
